# P-1702. Evaluation of Prescribing Practices and Treatment Failure in Obese Patients Diagnosed with Purulent Skin and Soft Tissue Infections in Outpatient Settings Across Michigan

**DOI:** 10.1093/ofid/ofae631.1868

**Published:** 2025-01-29

**Authors:** Mackenzie Miller, Destiny Hughson, Noah Blower, Andrew Jameson, Lisa E Dumkow

**Affiliations:** Trinity Health Grand Rapids, Grand Rapids, Michigan; Trinity Health Grand Rapids, Grand Rapids, Michigan; Trinity Health Grand Rapids, Grand Rapids, Michigan; Trinity Health Grand Rapids, Grand Rapids, Michigan; Trinity Health Grand Rapids, Grand Rapids, Michigan

## Abstract

**Background:**

Obesity has been associated with increased risk of antibiotic treatment failure in skin and soft tissue infections (SSTI), however optimal treatment of SSTIs in obesity is unclear with limited data available. The goal of this analysis was to assess prescribing practices and risk factors for treatment failure in obese patients treated for purulent SSTI with oral antibiotics in the outpatient setting.

**Methods:**

This retrospective, multicenter cohort study evaluated patients treated for purulent SSTI from February 1, 2020 - August 31, 2023, at Michigan outpatient locations within a health system. Patients ≥ 18 years with a body mass index (BMI) ≥ 30 kg/m2 and creatinine clearance ≥ 30 mL/min who received a ≥ 5-day course of oral antibiotics for purulent SSTI were included. Key exclusion criteria were chronic infections, antibiotic treatment within the past 30 days, and those requiring hospital admission. The primary endpoint was to describe prescribing practices. Secondary endpoints included comparing risk factors for treatment failure, revisit, or admission within 30 days between patients with treatment success (Tx-S) and treatment failure (Tx-F). Treatment failure was defined as a recurrence of infection or need for re-treatment within 30 days.

**Results:**

200 patients were included (Tx-S, n = 100; Tx-F, n = 100). Baseline characteristics were similar between groups. Patients received 11 different antibiotic regimens with 26 dosing variations. Cephalexin monotherapy was most commonly prescribed (30%); 67% of patients received MRSA-active therapy, however, no patient received linezolid. Treatment failure was similar between those appropriately dosed (46.4%) vs. under-dosed (54.4%) (p=0.256), those who received 5-7 days of therapy (47.1%) vs. 10-14 days (54.4%) (p=0.311), and those who received MRSA-active therapy (52.2%) vs. no MRSA therapy (45.5%) (p=0.367). Patients treated with clindamycin were more likely to experience treatment failure (73.7% vs 47.5%, p=0.030) and had a higher rate of 30-day revisit (68.4% vs 47%, p=0.075).

**Conclusion:**

Antimicrobial prescribing for outpatient treatment of purulent SSTIs in obese patients was heterogenous across a Michigan health system. Opportunities exist to optimize agent selection, dosing, and duration of therapy in this population.

**Disclosures:**

**All Authors**: No reported disclosures

